# Facial Palsy as Initial Symptom in Glycine Receptor Antibody Positive Progressive Encephalomyelitis With Rigidity and Myoclonus: A Case Report

**DOI:** 10.3389/fneur.2022.866183

**Published:** 2022-04-25

**Authors:** Li Wang, Rui Zhang, Kai Liu, Yafang Xu, Bo Song, Yuming Xu

**Affiliations:** ^1^Department of Neurology, The First Affiliated Hospital of Zhengzhou University, Zhengzhou, China; ^2^Henan Key Laboratory of Cerebrovascular Diseases, Zhengzhou, China

**Keywords:** progressive encephalomyelitis with rigidity and myoclonus, glycine receptor antibody, facial palsy, autoimmune disease, complications, case report

## Abstract

Progressive encephalomyelitis with rigidity and myoclonus (PERM) is a rare and disabling syndrome characterized by painful spasms, myoclonic jerks, hyperekplexia, brainstem signs, and dysautonomia, which is considered to be a severe form of stiff person spectrum disorder (SPSD) and is mostly associated with glycine receptor antibodies. The PERM has an acute or subacute course, with complex and varied initial symptoms mainly manifest as stiffness and pain. The authors present the case of a male patient admitted for intractable stiffness and paroxysmal myoclonus of the lower extremities preceded by a 5-day history of facial weakness. After admission, his symptoms deteriorated rapidly. He developed progressive generalized hypertonia and painful spasms, which quickly spread to the upper extremities, and he suffered frequent paroxysmal myoclonus. Serum and cerebrospinal fluid (CSF) were tested by a cell-based assay, and both were positive for glycine receptor antibodies (GlyR-Abs). The patient developed complications, such as crushed teeth, lumbar vertebral compression fractures, and psoas major muscle abscess, during rapid disease progression, although he responded well after being treated with intravenous methylprednisolone and immunoglobulin. This report of PERM, initiated as facial palsy followed by acute progression, helps to expand the clinical spectrum of this rare autoimmune disorder and raise awareness of the prevention of complications.

## Introduction

Stiff person spectrum disorder includes a group of immune-mediated disorders characterized by fluctuating muscle rigidity and painful spasms with pronounced stimulus sensitivity. The PERM is a more severe disease form of SPSD-exhibiting brainstem symptoms, long tract signs, and additionally autonomic features and is mostly associated with GlyR-Abs. The PERM has complex initial symptoms, which mainly present as limb stiffness and pain or brainstem symptoms, such as oculomotor disturbance, nystagmus, ptosis, and bulbar symptom (1). The report of facial palsy as an initial manifestation is rare. We report a case of acutely progressed PERM that led to severe complications, although responded well to immunotherapy. The GlyR-Abs were found positive both in CSF and in serum. No evidence for an underlying systemic neoplasm was found.

## Case Presentation

**Figure 1:**
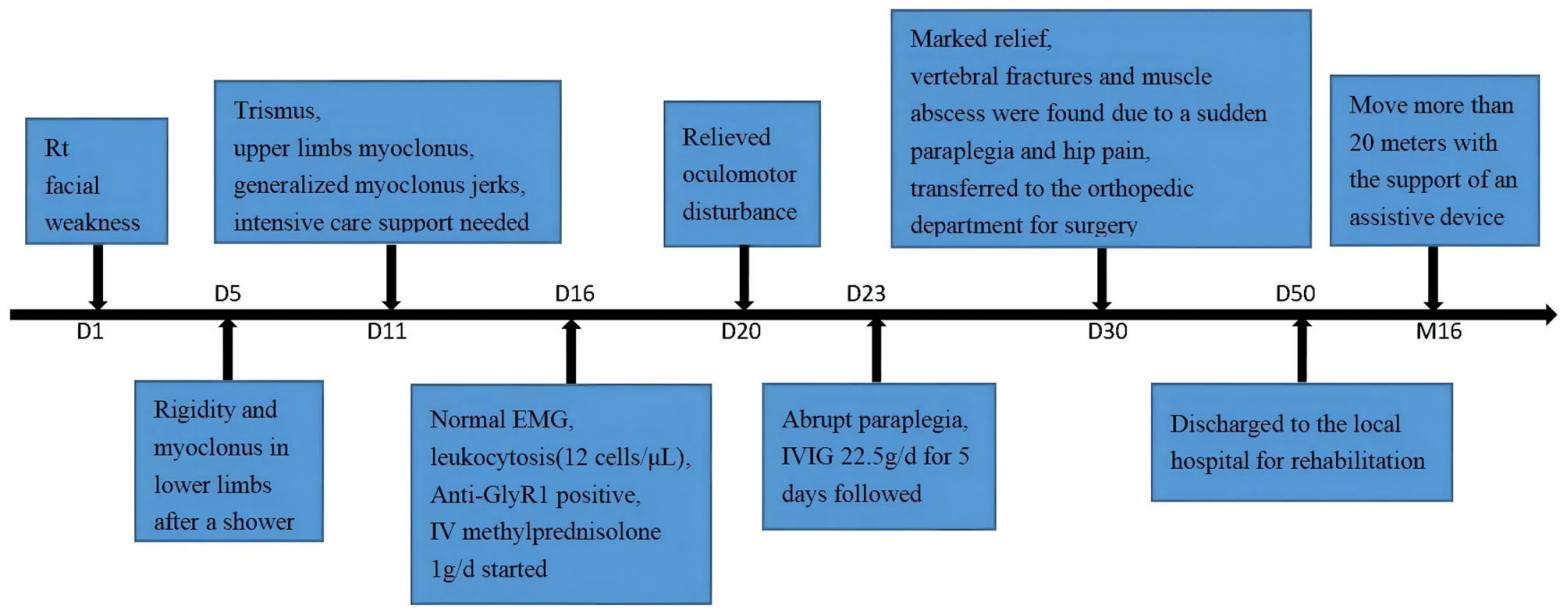
Timeline with diseases' onset and course.

A 61-year-old previously healthy male baker was admitted to the hospital for left-sided facial weakness (Figure 1). Neurological examination revealed lower motor neuron facial weakness of the left side without other abnormalities. The brain MRI showed lacunar infarction, and it was treated as a stroke. Treatments to improve cerebral circulation, anti-platelets, and lower lipids were tried, but his symptoms did not resolve, and the patient gradually developed right-sided ptosis during hospitalization. He did not complain of another discomfort. However, 5 days after the onset, the patient suddenly developed rigidity and intermittent involuntary tic-like jerks in the left lower extremity after returning home to take a shower. This rigidity and painful spasm expanded to the right-side lower extremity the next day. Pregabalin and cotrimoxazone were administrated without much relief, and the rigidity continued to deteriorate in the following 5 days, rendering a walking disability. The clinicians had no clue of his condition, so he was referred to our department.

On admission, he was well oriented. Neurological examination revealed contracted muscles in the spine and lower extremities; his spine was rigid and his ankle joints were fixed in a hyper-extended position. Paroxysmal painful myoclonic jerks of lower extremities were observed (Supplementary Video 1). He also had multiple cranial nerve dysfunctions: insufficient abduction, upward gaze diplopia, horizontal-gaze-evoked nystagmus of the left eye, eyelid ptosis, upward gaze paralysis of the right eye, and left-sided facial palsy. Also, petechiae on the left lateral calf were observed. The patient had a 20-year history of smoking and alcohol abuse. Trauma, infection, poisoning, drugs, psychiatric disease, and family genetic history were denied. He had no history of being scratched or bitten by dogs or cats. No insect bites were found.

Laboratory testing revealed creatine kinase: 1,600 U/L, creatine kinase isoenzyme: 111.5 U/L, lactate dehydrogenase: 1,578 U/L, white blood count, 20,000/mm^3^ with neutrophil at 84.6%, lymphocytes at 7.4%, monocytes at 7.7%, hemoglobin: 15. g/dl, platelet counts: 173/mm^3^, C reactive protein, 17.3 mg/L, IgE antibody, 393 IU/ml (0–100), granular type +, antiRo52 +, and HbeAb +, HBcAb +. Biochemical investigations, including liver and kidney function tests, thyroid function, blood glucose, blood ammonia, serum electrolytes, blood clotting function, tumor biomarkers, folate, vitamins B1 and B12, homocysteine, and electrolytes, were normal.

Clusters of herpes appeared in his mouth the night he was admitted. His symptoms deteriorated rapidly since admission; the rigidity and spasms spread to the upper extremities and masseter muscles on the next day, so he was transferred to the intensive care unit (ICU). He suffered frequent bouts of painful myoclonic jerks triggered by touch and spontaneously lasting about 30 s with an aura in the form of hallucinations and fear. He also exhibited emotional irritability and anxiety, as well as autonomic dysfunction, such as episodes of fever, tachycardia, and hypertension. Increased doses of diazepam (20 ml/h) and dexmedetomidine (10 ml/h) were administered only to cease the symptoms transiently. The rigidity was so severe that it was impossible to bend his knee or ankle joints passively, and some of his teeth were broken due to intense masticatory spasms. Based on the typical clinical features (the rapid onset, brainstem involved, and movement disorder), infectious or autoimmune encephalitis was considered as a high possibility.

Examinations were performed under anesthesia. The CSF examination revealed mild leukocytosis (12 lymphocytes/μL) (0–5 cells/μl), lymphocyte: 76%, mononuclear cell: 20%, plasma cells: 1%, protein: 355.7 mg/L (150–450 mg/L). The CSF cultures were negative for bacteria, tubercle bacillus, and fungi. No abnormalities in tumor markers were found. Brain MRI only showed a slightly high-signal on brainsteming T2/fluid-attenuated inversion recovery (FLAIR) (same with previous). Meanwhile, anti–GlyR antibodies were detected positive in serum (tilter 1:100) and CSF (tilter 1:100) through a cell-based assay. Antibodies for autoimmune encephalitis, including anti-GAD65, anti-Casper2, anti-DPPX, anti-mGluR1, anti-GABAB, anti-DRD2, anti-NMDA, anti-LGI1, anti-Neu3a, anti-AMPA1, anti-AMPA2, anti-IgLON5, and anti-mGluR5, were negative. Anti-GlyR antibody-associated PERM was suspected, and we started the intravenous methylprednisolone (Day 6 after admission) (1,000 mg/d and halve it every 3 days to an oral dose). (Day 6 after admission).

**Figure 2:**
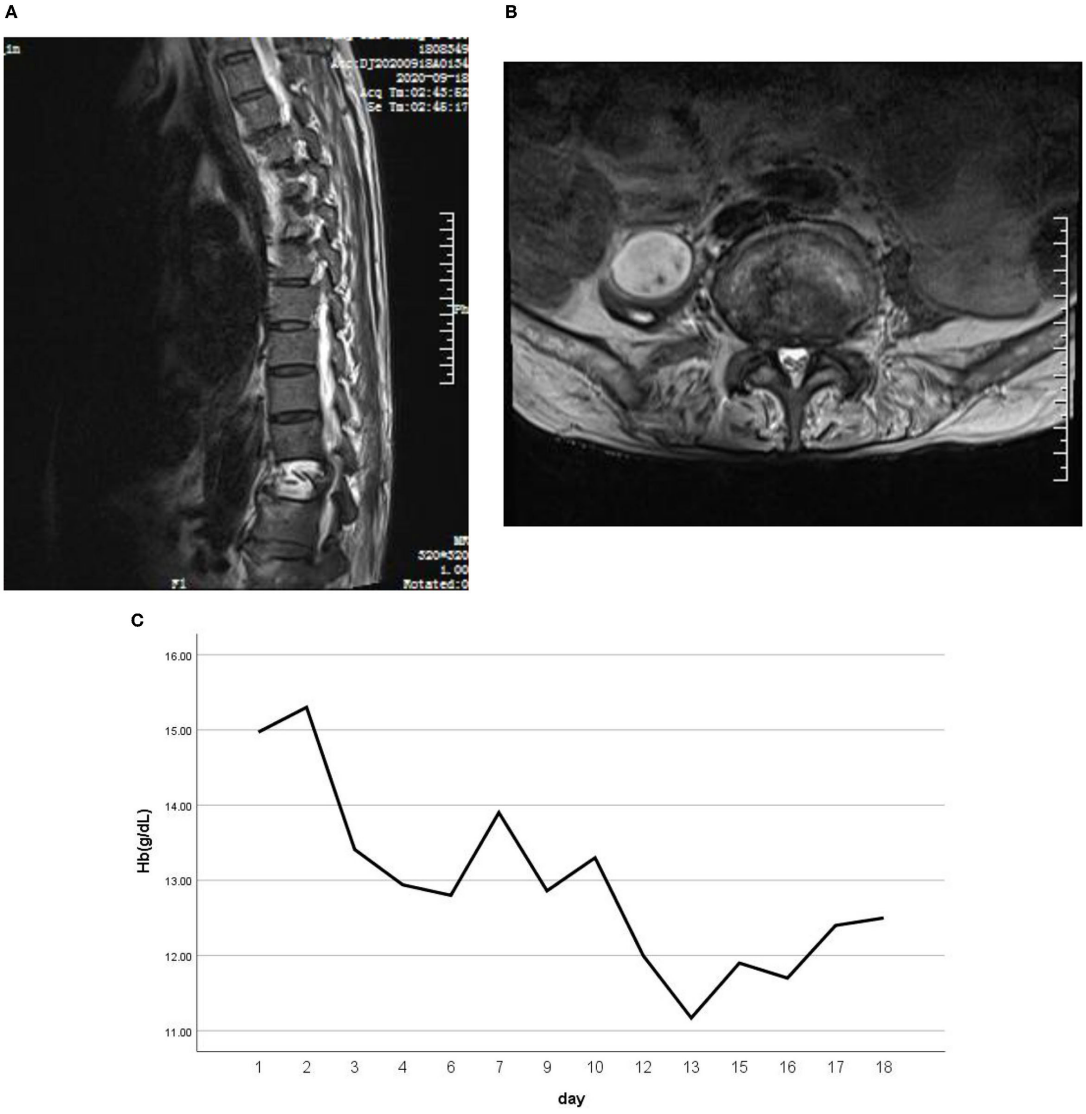
Lumbar MRI showed an oval-shaped confined mass in the right-side psoas major muscle **(A)**. **(B)** T4/5/7/12 compression fractures. **(C)** A blood routine test suggested progressive anemia.

He showed transient remission with the recovery of oculomotor function and reduction of myoclonus bouts after 8 days of therapy of IV methylprednisolone. However, there was sudden paraplegia on the 9th day, while spasms and myoclonus were still severe; 5-day IV immunoglobulin (0.4 g/kg/d) was followed. He showed marked improvement, with only paroxysmal bouts of masticatory spasms remaining. Since the generalized pain was relieved, he complained of unbearable pain in his back and hip. Lumbar MRI showed an oval-shaped confined mass in the right-sided psoas major muscle and T4/5/7/12 compression fractures (Figure 2). The mass was confirmed to be an abscess by an ultrasound-guided puncture, and *Streptococcus constellatus* infection was proved by bacteria culture. The patient was transferred to the orthopedic department.

## Follow-up and Outcome

After discharge, the methylprednisolone was gradually reduced orally, and mycophenolate mofetil was prescribed. He was symptomatic for transient masticatory spasms, lasting about 1 s when blown by the wind or in emotional instability at 12-month follow-up, and the twitching disappeared 2 months later, but he still had a mild left-side facial weakness. After a year-long rehabilitation, the strength of the patient's lower limbs improved a bit, and he was able to move 20 m with the support of an assistive device.

Written informed consent was obtained from the participant for the publication of this case report.

## Discussion

The PERM is a variant of stiff person syndrome, usually presenting with classical symptoms of SPS (axial and limb stiffness, painful muscle spasms) associated with myoclonus, hyperekplexia, brainstem signs, pyramidal signs, dysautonomia, and cognitive impairment with an aggressive course (1). GlyR-Abs was firstly reported to be related to PERM in 2008 by Hutchinson et al. (2), and they were most frequently found in patients with PERM (3). Glycine receptors are pentameric ligand-gated chloride channels present mainly in the adult brainstem and spinal cord, facilitating inhibitory signaling, and the stoichiometry has been recently proved to be 4 alpha: 1 beta subunits (4). Mutations of GlyRα1 result in hereditary hyperekplexia, a disorder characterized by excessive startle responses in infancy, which resembles the phenotype of PERM (5). Auto-antibodies against GlyRα1 show pathogenic characteristics *in vitro* such as complement activation and receptor internalization (1). Another study found that glycinergic currents are greatly disrupted by short incubations in patient IgG at room temperature, which suggests that the pathogenic mechanisms include direct antagonistic actions on glycine receptors (6). Whatever the mechanism is, the impairment of the GlyRs on the brainstem nuclei of spinal inhibitory interneurons may cause continuous firing of motor neurons, leading either to myotonia of the encephalomyelitis and rigidity seen in PERM.

Initial presentation of PERM can be very unspecific; 25% of patients may present with brainstem symptoms, such as oculomotor disturbance, nystagmus, ptosis, and bulbar symptom. Patients, then, progress acutely or subacutely to characteristic muscle stiffness and spasms; some may lead to death in the acute phase (1). Several reports mentioned facial weakness during disease progression, among which both lower motor neuron facial weakness and upper motor neuron weakness were described (7–13). Anti-GlyR1 is believed to be responsible for excessive muscle activation since it disrupts the function of inhibitory interneurons. However, the exact mechanism for cranial nerve palsy is not clear. Since congenital bilateral vocal cord paralysis has been experimentally shown to be associated with impaired glycine neurotransmission (14), we assume that they may share a similar pathophysiological mechanism. Neuron damage due to blood-brain barrier disruption or other potentially unknown pathogenic antibodies may play a role. A typical PERM presented 5 days post symptom initiation as facial palsy is unique and surely expands the clinical spectrum of PERM.

After the atypical onset, the patient developed typical PERM with an acute course. Although responded well to immunotherapy, he suffered skeletal fractures and teeth breakage due to severe spasms. A skeletal fracture happens in patients with SPSD (15, 16). It is difficult to discern whether hormonal shock therapy played a role in the fractures, but prompt differential diagnosis and timely intervention would certainly benefit, especially when the patient presents with an acute disease course on the basis that patients with GlyR-Abs usually show substantial and sustained improvement with immunotherapy (7). Besides, more thought should be given to the choice of treatment modality to avoid complications such as fractures in patients who are old-aged and have severe acute muscle contractions. Neurologic disorders, such as metabolic encephalopathies, himoto encephalopathy, tetanus, Isaacs' syndrome, malignant catatonia, and serotonin syndrome that manifest similar clinical presentations, should be considered as differential diagnoses. The medication history, blood tests, CSF findings, and brain MRI, especially auto-antibodies, are essential for differentiation.

The trigger of the autoimmune response is unknown. Tumors such as thymoma and Hodgkin's lymphoma are documented in approximately 20% of patients with PERM (17). In this case, the patient had no evidence of malignancy or thymoma on a CT scan. It is reported that the human herpes simplex virus (HSV) can trigger autoimmune encephalitis (18, 19). The patient experienced an attack of HSV, which lasted about 20 days. However, it is unknown whether the HSV infection acted as a trigger or a consequence of impaired immunity since no virological examination of CSF was performed. Besides, comorbid autoimmune diseases were not uncommon among patients with GlyR-Abs 13 of 45 that had another autoimmune condition (1). Although there were auto-antibodies like Ro52 and granular type detected positive, the patient did not have a confirmed diagnosis.

The abscess of the psoas major muscle with *Streptococcus constellatus* cultivated positive is interesting. *Streptococcus constellatus* is a subgroup of viridans streptococci widely distributed in the oral cavity, nasopharynx, gastrointestinal tract, and vagina (20). These bacteria have the capability of causing pyogenic infections and abscess formations mainly in the respiratory tract, brain, liver, bone, and soft tissues on a rare occurrence (21). So, how did this patient's abscess of the psoas major muscle arise? Rui Shimazaki reported a case of PERM with anemia, complicated with bilateral iliopsoas hematomas (22), and he speculated that a simple microtrauma due to isometric muscle contraction could potentially result in muscle and capillary tears, subsequently leading to spontaneous muscle hematomas. He also suggested a survey for intramuscular hematoma, including iliopsoas hematoma, when progressive anemia is present in patients with PERM. Our patient actually did have progressive anemia (Figure 2C). Therefore, it can be boldly speculated that the lumbar muscle abscess is an opportunistic pathogenic infection based on a hematoma caused by muscle contraction. This case illustrates that patients with PERM may develop multiple complications, among which some are unpredictable.

## Conclusion

The PERM is a rare autoimmune disorder with complex symptoms. Patients may present an acute and severe disease course and lead to a variety of complications. Recognizing the atypical initial presentations of PERM is important for patients with an acute course for fast recognition and proper treatments are essential to prevent irreversible damage.

## Data Availability Statement

The datasets presented in this article are not readily available because of ethical and privacy restrictions. Requests to access the datasets should be directed to the corresponding author/s.

## Ethics Statement

Written informed consent was obtained from the individual(s) for the publication of any potentially identifiable images or data included in this article.

## Author Contributions

LW and RZ wrote the manuscript. KL, RZ, and BS examined and treated the patient. YaX and YuX participated in revising the manuscript. All authors contributed to the article and approved the submitted version.

## Conflict of Interest

The authors declare that the research was conducted in the absence of any commercial or financial relationships that could be construed as a potential conflict of interest.

## Publisher's Note

All claims expressed in this article are solely those of the authors and do not necessarily represent those of their affiliated organizations, or those of the publisher, the editors and the reviewers. Any product that may be evaluated in this article, or claim that may be made by its manufacturer, is not guaranteed or endorsed by the publisher.

## Supplementary Material

Supplementary Video 1 Broken teeth and difficulty opening the mouth due to facial muscular spasms and unhealed herpes on the tongue and around the mouth. Fixed ankle joints and upper extremities shaking. A bout of myoclonic jerks in lower extremities.
